# Tunable parity-time symmetry vortex laser from a phase change material-based microcavity

**DOI:** 10.1038/s41378-023-00622-z

**Published:** 2023-11-10

**Authors:** Ying Su, Hongji Fan, Shitong Zhang, Tun Cao

**Affiliations:** 1 School of Optoelectronic Engineering and Instrumentation Science, Dalian University of Technology, Dalian, 116024 China; 2 School of Science and Letters, UC Davis, 2100 5th St, Davis, CA 95618 USA

**Keywords:** Nanocavities, Nanophotonics and plasmonics

## Abstract

Traditional light sources cannot emit an electromagnetic (EM) field with an orbital angular momentum (OAM), limiting their applications in modern optics. The recent development of the OAM laser, mainly based on micro- and nanostructures, can satisfy the increasing requirements for on-chip photonics and information capacities. Nevertheless, the photonic structures have fixed parameters that prevent these OAM lasers from being dynamically tuned. Here, we propose tunable vortex lasing from a microring cavity integrated by a phase change material, Ge_2_Sb_2_Te_5_ (GST225). By modulating the complex refractive index to create an exceptional point (EP) to break the degeneracy of whispering gallery modes with opposite orientations, the microlaser working at the EP can impart an artificial angular momentum, thus emitting vortex beams with well-defined OAM. The grating scatter on the edge of the microring can provide efficient vertical radiation. The vortex laser wavelength from the GST225/InGaAsP dual-layered microring cavity can be dynamically tuned by switching the state of GST225 between amorphous and crystalline without changing the microring geometry. We construct an electric-thermal model to show the tuning range of operating wavelengths (EPs) from 1544.5 to 1565.9 nm in ~25 ns. Our study on high-speed tunable PT-symmetry vortex lasers facilitates the next generation of integrated optoelectronic devices for optical computing and communications in both classical and quantum regions.

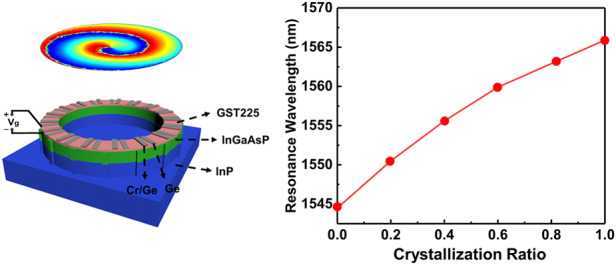

## Introduction

Modern optical communication explores electromagnetic (EM) wave features to enhance the bit rate per unit carrier frequency. Conventional methods exploit phase and polarization diversity^1^. Recently, the optical vortex carrying orbital angular momentum (OAM) $l\hslash$ has provided an extra possibility for improving the spectral efficiency^2,3^, where $l$ is an arbitrary integer called topological charge and ${\rm{\hslash }}$ is the reduced Plank constant^4^; moreover, OAM possesses a topological phase singularity at the beam axis in which the field intensity disappears owing to the undetermined phase^1,5^. Since there is no limit on the OAM order, the vortex beams can be applied to encoding information in both classical^3,6^ and quantum systems^7,8^. Moreover, the null light intensity in the beam center can be used to obtain super-resolution imaging^9^. Furthermore, optical vortices are promising candidates for laser manipulation^10^, optical measurement^11,12^, optical tweezers^13^, digital imaging^14^ and optical spanners^15^. These applications need controllable OAM and frequency tunability for versatility with optical vortex sources. In this study, we propose a strategy for an electrically biasing microring laser based on phase change materials (PCMs) with the ability to tune the working wavelength while maintaining toroidal intensity distributions.

Initially, OAM laser beams were generated by transmitting free-space light without OAM through forked linear gratings or spiral phase plates^1^. Other techniques exploited spatial light modulators^16^, $q$ plates^17,18^, metasurfaces^19,20^, Archimedean spiral-shaped waveguides^21^, phased array nanoantennas^22^, and notched microring resonators coupled to a bus waveguide^23^. However, the light source and the OAM generator are separate elements in these schemes. A monolithic, compact laser chip with nondegenerate modes exhibiting nontrivial OAM can increase efficiency, reduce cost, and provide greater integration. To this end, recent studies have demonstrated that breaking the parity-time (PT) symmetry at an exceptional point (EP) is a feasible approach to obtain unidirectional laser emission from the microring cavity^24–28^. By adequately introducing spaced loss and gain segments along the microring, the coupled modes in the structure share the same eigenfrequency but have opposite modal loss/gain coefficients; this can break the PT symmetry and produce a single-mode microring laser^29,30^. These approaches, however, apply to nontunable vortex sources; a design method with dynamic control of the OAM mode in the microring cavity is needed.

Recently, chalcogenide PCMs have been integrated into nanophotonic devices to achieve tunable spectra^31–38^, as initiated by Ovshinsky^39^. The spectral change is caused by the pronounced changes in the optical characteristics of the chalcogenide semiconductor in the amorphous and crystalline states. This drastic change is caused by a significant variation in the chemical bonding on the phase transition between the amorphous and crystalline phases^40^. This unique property enables the dielectric constant to greatly vary, thus dynamically engineering the working frequency of the devices; thus, the chalcogenide semiconductor is a promising candidate for use in nonvolatile, rewritable data storage with high cyclability, fast tuning speed, great scalability, and thermal resistance^41,42^. Until now, the chalcogenide semiconductor has been used in many kinds of elements for on-chip photonic integration, apart from laser chips.

In the following, we propose a cavity design that can vertically radiate tunable OAM beams using a Ge_2_Sb_2_Te_5_ (GST225)/InGaAsP dual-layered microring cavity, where the GST225 is a typical chalcogenide semiconductor. An EP is created and supports a single-mode vortex laser that can precisely determine the topological charge of the OAM mode by periodically placing Ge and Cr/Ge patches on the GST225/InGaAsP microring along the circular direction. This mimics complex refractive index changes. Rather than changing the microring cavity geometry, the operating wavelength can be actively modulated on demand. A photothermal model shows that crystallization and re-amorphization phase transitions can be attained in 25 and 5 ns, resulting in a dynamically tunable vortex laser. Our concept is constructive for a class of non-Hermitian photonics devices where the continuous tuning of PT symmetry vortex laser is significant.

## Results and discussions

**Fig. 1:**
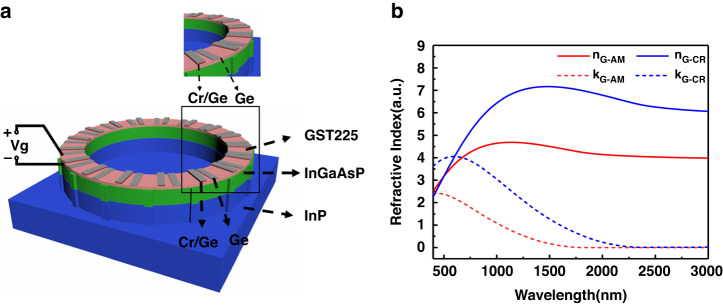
The microring cavity resonator schematically and the complex refractive index of GST225. **a** Schematic of the GST225/InGaAsP dual-layered OAM microring laser on the InP substrate. The diameter, width, and height of the ring cavity are 4000, 250, and 650 mm, respectively. The heights of InGaAsP and InP are 600 and 800 nm, respectively. The Cr/Ge bilayer with a thickness of 5 nm/11 nm and the Ge single layer with a thickness of 13 nm are periodically located in the azimuthal direction above the GST225/InGaAsP/InP microring, which mimics the gain/loss and real index parts of an EP modulation at$\,{{n}^{\prime} }={{n}^{{\prime}{\prime}}}=$ 0.01 to allow unidirectional power circulation. There are 16 pairs of Cr/Ge bilayers and Ge single layers. The equidistant sidewall scatterers with a value of $w=$ 17 on the microring periphery couple the lasing emission upward, generating the OAM vortex emission with a helical wavefront. The topological charge is described by $\,p=w-m=$ 1. **b** VASE measurement of the complex refractive index of ${n}_{G}$ (solid lines) and ${k}_{G}$ (dashed lines) for both crystalline (blue lines) and amorphous (red lines) phases of GST225

We consider a microring cavity resonator schematically shown in Fig. 1a. The cavity is based on an InGaAsP gain material system that can be applied to the C-band of optical communication^43^. Notably, the microring cavity can support the whispering gallery mode (WGM) that carries significant OAM. However, the mirror symmetry of the cavity produces both counterclockwise and clockwise eigen-WGMs that coexist in pairs inside the cavity, causing a cancellation of their OAMs^27^. To achieve the OAM of an individual WGM, an efficient selection mechanism of either counterclockwise or clockwise modes is vitally needed. Conventional bulk optics showed that the unidirectional ring laser was achieved by placing a nonreciprocal isolator in the light path^44^. The unidirectional energy flow was enabled by the isolator, which caused nonreciprocally counterpropagating lights. Nevertheless, this technique is not applicable at the micro- and nanoscale since it is a formidable challenge to achieve microscale isolators^26^.

The recent development of PT symmetry has provided another promising approach for obtaining unidirectional power circulation by modulating gain and loss to form an exceptional point (EP) at which multiple eigenstates coalesce into one^45,46^. Therefore, herein, we use the EP to achieve OAM laser emission. The microring resonator is made by depositing a 50 nm thick GST225 and 600 nm thick InGaAsP dual layers onto an InP substrate. The refractive indices of InGaAsP and InP are 3.42 and 3.17, respectively^26^. The grating with a complex refractive index is designed with alternate bilayer Cr/Ge and single-layer Ge patches periodically placed above the GST225 along the azimuthal direction ($\theta$); this corresponds to the modulations of gain/loss (${{n}^{\prime\prime}}$) and the real part index (${n}^{{\prime} }$) in the cavity, respectively, which explained by the refractive index Equations S1 in the Supplementary Information.

Assuming a slight change in the index variation and insignificant scattering loss in the PT microring resonator, we express the coupled mode equations for the desired WGM order as follows^26^:

1 $$\left(\begin{array}{c}\frac{dA}{Rd\theta }\\ \frac{dB}{Rd\theta }\end{array}\right)=\left(\begin{array}{cc}i{k}_{0} & i\left(n^{\prime} +{n^{\prime\prime}} \right)\kappa \\ i\left(n^{\prime} -{n^{\prime\prime}} \right)\kappa & i{k}_{0}\end{array}\right)\left(\begin{array}{c}A\\ B\end{array}\right)$$

where $A={A}_{0}{e}^{{ikR}\theta }$ and $B={B}_{0}{e}^{{ikR}\theta }$ and both represent the clockwise and counterclockwise propagating harmonic waves, respectively, $R$ the microring resonator radius, ${k}_{0}$ the wavenumber of the modes in the unmodulated ring resonator, and $\kappa$ the coupling between the counterclockwise and clockwise modes. By solving the coupled mode equations originating from the modulations of the complex index and gain/loss presented in Equation S1, we derive the WGM complex wavenumber, as follows:

2 $$k={k}_{0}\pm \kappa \sqrt{{n^{\prime}}^{2}-{n^{\prime\prime}}^{2}}$$

The coupling between the two modes disappears at $\,{{n}^{\prime} }={{n}^{\prime\prime}}$. As a result, an EP occurs; two WGMs with counterpropagating directions coalesce and unexpectedly degenerate. A unidirectional laser shows strong emission, which is explained by semiconductor rate equations in the Supplementary Information. Therefore, the azimuthally continuous phase evolution causes the counterclockwise WGM with a large OAM to unidirectionally circulate in the cavity^28^. The grating implanted along the microring perimeter extracts the OAM related to the unidirectional power oscillation upward into free space. It was shown that only by satisfying the angular phase-matching condition can induce a WGM emitting into a free-space beam^26^,

3 $${\eta }_{rad}=m-w$$

where $m$ and $w$ are the azimuthal order of the targeted WGM and the sidewall equidistant scatterers around the microring resonator, respectively. The negative integer $m$ corresponds to the opposite propagating direction of the WGM. The ${\eta }_{{rad}}$ phase shift per unit azimuthal angle, or azimuthal propagation constant of the emitted light, provides the OAM-carrying wave vector’s azimuthal component. The propagation constant of the radiation mode along the z-direction is given by the following:

4 $${k}_{{rad},z}=\sqrt{{(2\pi /\lambda )}^{2}-{({\eta }_{{rad}}R)}^{2}}$$

where $\lambda$ is the free-space wavelength. The topological charge $p=m-w$ of the scalar vortex is associated with the OAM of the vector vortex. The emitted light carries an OAM of $p{\rm{\hslash }}$, where ${\rm{\hslash }}=h/2\pi$ and $h$ is Plank’s constant. The protruding grating can only couple the WGM into the free-space vortex beam mode with a certain OAM index $p$ since the extra vortex beam that does not satisfy Eq. 1 cannot couple into free space. Thus, we have an extremely simple yet efficient single-mode OAM emitter strategy, where $p$ takes an integer number depending on the variation between integers $m$ and $w$. Equation S1 shows that the light trapped in an ${mth}$ -order WGM (with an OAM of $m{\rm{\hslash }}$ per photon^16^) is diffracted by the angular grating and escapes into free space, altering its OAM by $w{\rm{\hslash }}$ per photon along the way. Once the microring cavity is fabricated, $w$ is fixed; however, $m$ can be varied by exciting the selected WGM. Thus, altering the cavity refractive index tunes the cavity resonance, alters the OAM and provides tunable vortex lasing. Based on this concept, we propose a tunable OAM emission from a PT symmetry microring cavity, where the frequency of OAM (cavity resonant frequency) is modulated by switching the GST225 state between crystalline and amorphous.

The real, ${n}_{G}$ (solid lines) and imaginary, ${k}_{G}$ (dashed lines) parts of the complex refractive index of ${N}_{G}={n}_{G}+i\,\times \,{k}_{G}$ for a planar GST225 layer with a thickness of 50 nm for the amorphous (red lines) and crystalline (blue lines) phases are presented in Fig. 1b. The ${N}_{G}$ was determined by variable angle spectroscopic ellipsometry (VASE). A Tauc-Lorentz model was fitted to the measured data. The sharp change in ${N}_{G}$ produced tunable microring cavity resonances. The refractive index changes of GST225 originated from a transition from mainly covalent bonds in the amorphous phase to resonant bonds in the face-centered cubic crystalline phase^41^. This unusual optical property was realistic, recognized and mainly utilized for data storage devices. Notably, the reversible phase change in GST225 was highly reproducible and experimentally demonstrated in data storage devices for a billion cycles^47,48^. Thus, GST225 alloys have recently been applied to the field of tunable nanophotonics^35−38^.

A plane-wave source is normally used to illuminate the cavity. Stable and efficient single-mode lasing with good sideband suppression is achieved by applying EP modulation to the unidirectional power stream of the OAM microlaser. The peak resonance corresponds to an OAM lasing mode and can be calculated with the following equation:

5 $${\lambda }_{{\rm{m}}}=\frac{2\pi {n}_{e}R}{m}$$

where a targeted WGM has a specific azimuthal order $m$, radius $R$ and equivalent refractive index ${n}_{e}$. Here, ${n}_{e}$ changes with the phase change of GST225 from 2.25 to 2.28. Based on Eq. 5, the resonant peak shifts to a longer wavelength (from 1544.5 nm to 1565.9 nm) owing to the phase change of GST225 between crystalline and amorphous. The quality ($Q$) factor of the microring cavity retains its large value of ~37500. Thus, this cavity is a potential candidate for a single mode OAM laser operation that can be tuned.

**Fig. 2:**
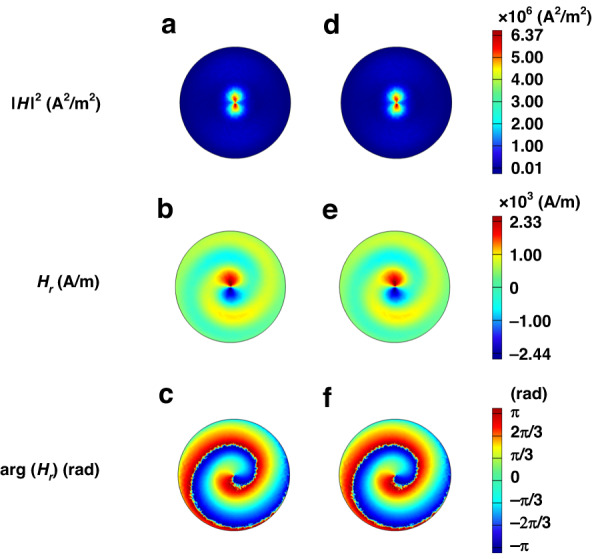
The simulations of the vortex laser emission at the resonant wavelength. **a**–**c** Vortex laser emission from the ring cavity at λ = 1544.5 nm for the amorphous phase. **a** Cross section of the magnetic field intensity ${{\rm{|}}H{\rm{|}}}^{2}$ in the cavity. **b** Cross section of the radial component of the magnetic field ${H}_{r}$ in the vortex beam. **c** Phase distribution of the emitted magnetic field (arg(${H}_{r}$)) along the cross section at the far field (*z* = 4800 nm), showing a clear OAM charge-one vortex. **d**–**f** Vortex laser emission from the ring cavity at $\lambda =$ 1565.9 nm for the crystalline phase. **d** Intensity distribution of ${{\rm{|}}H{\rm{|}}}^{2}$ in the cavity cross section. **e** Cross section of the radial component of ${H}_{r}$ in the vortex beam. **f** Phase distribution of arg(${H}_{r}$) at $z=$ 4800 nm

Notably, the index modulation achieved by the paired bilayer Cr/Ge and single-layer Ge gratings causes the cavity to form an EP; moreover, the protrudent scatters cannot change the EP due to its fixed number $w=$17. Therefore, we can vary the operating frequency by changing the GST225 state while maintaining the ring cavity at the EP. Figure 2a−c shows the simulations of the vortex laser radiation at the resonant wavelength $\lambda$ of 1544.5 nm for the amorphous phase. The refractive index modulation leads to the EP, at which two counterpropagating WGMs coalesce to a counterclockwise WGM ($m=$16) circulating inside the cavity (Fig. 2a). Notably, the center of the WGM mass moves outward due to the finite curve of the microring (centrifugal force), and the modulations introduced along the microring boundary have a sensitive impact on the WGM. Thus, we place the scatterers along the outer edge of the ring to efficiently extract the OAM lasing. The scatterers extend from the outer perimeter by $\Delta R=$6 nm, and the scatter angular width is ${{\rm{\delta }}}_{\theta }=$ 0.03 in radians. They are equidistantly located around the outer edge of the cavity. The WGM ($m=$16) can couple with a vertically radiating vortex beam with specific OAM ($p=$1) by the scatterers, and the OAM lasing mode radiates the vortex beam into free space (Fig. 2b). On one complete circle around the vortex center, the phase of the electric (*E*-) field varies by 2π. The light axis has a topological phase singularity point where the phase is disrupted at the center of the radiation path (Fig. 2c). The wavelength of OAM laser radiation can be tuned by switching the state of GST225 between crystalline and amorphous because the GST225 phase transition affects the refractive index of the ring cavity and thus the cavity resonance. Figure 2d–f shows the vortex laser emission from the ring cavity at $\lambda =$1565.9 nm for the crystalline phase. The field maps of the system are similar to those at $\lambda =$1544.5 nm (Fig. 2a–c), indicating that the unidirectional WGM with $m=$16 and OAM with $p=$1 can be excited to create a vortex beam. The homogeneous field intensity (${{\rm{|}}H{\rm{|}}}^{2}$) distributions of WGMs with $m=$16 for the amorphous (Fig. 2a) and crystalline (Fig. 2d) phases demonstrate ideal mode properties. This result indicates that the EP does not change with the GST225 phase.

The transverse distributions of the radial component ${H}_{r}$ (Fig. 2b, e) and the corresponding phase distributions arg(${H}_{r}$) (Fig. 2c, f) further show that the vortex beams scattered by the outer grating elements have OAM orders of $p=$1 for both the amorphous and crystalline phases. By changing the phase of GST225 from amorphous to crystalline or vice versa, we can achieve OAM laser emission with a wide range of wavelengths (from 1544.5 to 1565.9 nm).

Moreover, GST225 can be partially crystallized by creating intermediate phases, possessing regimes of both crystalline and amorphous states^49^. This unique characteristic is promising for a photonic device with continuous tunability. The change in the complex refractive index (or the crystallization level) is verified using infrared reflectance measurements of the switching regime. In Eq. 6, we present the complex refractive index of partially crystallized GST225 with the Lorentz‒Lorenz model^50^, which can be broadly tuned by applying the gate voltage, ${V}_{g}$:

6 $$\frac{\varepsilon \left(\omega \right)-1}{\varepsilon \left(\omega \right)+2}=j\times \frac{{\varepsilon }^{{CT}}\left(\omega \right)-1}{{\varepsilon }^{{CT}}\left(\omega \right)+2}+(1-j)\times \frac{{\varepsilon }^{{AM}}\left(\omega \right)-1}{{\varepsilon }^{{AM}}\left(\omega \right)+2}$$

where ${\varepsilon }^{{AM}}\left(\omega \right)$ and ${\varepsilon }^{{CT}}\left(\omega \right)$ represent the complex refractive index of GST225 in the amorphous and crystalline phases, respectively, and$\,j$ is the crystallization ratio. To show an actively tunable PT symmetry vortex lasing, a few more calculations are carried out to continuously cause resonance in the ring cavity; this tunes the OAM mode by partially crystallizing the GST225 layer.

**Fig. 3:**
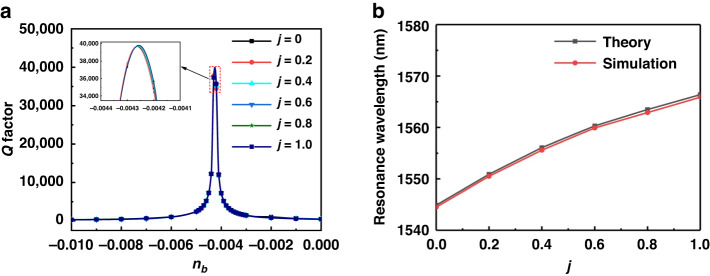
The gain effect in the vortex beam laser device at different crystallization ratios. **a** Spectra of the background gain-dependent cavity quality factor at various $j$. Increasing ${n}_{b}$ is used to mimic the uniform pumping produced by a gain-increasing process of InGaAsP microring. The $Q$ factor is ~365 for the ring cavity without gain. The $Q$ factor increases with the gain coefficiency by orders of magnitude, showing that the loss is compensated by the gain. Moreover, the inset shows a detailed enlarged image of the peak. **b** Characteristic frequency of the microring laser at various $j$. The vortex beam laser is at the EP (${n}^{{\prime} }={n}^{\prime\prime}=$ 0.01) with crystallization ratios of $j=$ 0, 0.2, 0.4, 0.6, 0.8, and 1. The theoretical value is calculated by Eq. 5, and the simulated value is obtained by COMSOL Multiphysics simulation

The propagating WGM is lossy owing to absorption and radiation. However, this can be compensated by the gain material (InGaAsP) under uniform pumping, and the lasing OAM mode emits vortex waves into the air space. Figure 3a shows the 3D full wave (COMSOL) modeling of the gain effect in the vortex beam laser device at the EP (${n}^{{\prime} }={n}^{\prime\prime}=$ 0.01) at crystallization ratios of $j=$ 0, 0.2, 0.4, 0.6, 0.8 and 1. The gain improvement of the microcavity is achieved by uniformly pumping the InGaAsP ring. In the model, this feature is replicated by enlarging the imaginary part of the background refractive index ${n}_{b}$. The quality ($Q$) factor is approximately 365 for different $j$ values in the cavity without gain (${n}_{b}=$ 0). The $Q$ factor significantly increases when the gain coefficiency increases, indicating that the gain compensates for the loss.

Thus, the OAM mode turns into a lasing mode and radiates the vortex beam. Notably, the OAM mode is lasing at different ${n}_{b}$ for different $j$. We then calculate the resonant frequency of the microring cavity at different $j$ by using Eq. 5. Figure 3b clearly shows that changing $j$ allows direct control over the resonant frequency of the microring laser. Herein, ${n}_{e}$ is 2.249, 2.258, 2.265, 2.272, 2.276, and 2.280 at $j=$ 0, 0.2, 0.4, 0.6, 0.8, and 1, respectively. As shown in Fig. 3a, the peak $Q$ factor of the cavity is maintained at approximately 37500 during the crystallizing process. In Figure S1 of the Supplementary Information, we show ${{\rm{|}}H{\rm{|}}}^{2}$, ${H}_{r}$, and arg(${H}_{r}$) of the microring laser at the different crystallization ratios, $j=$ 0, 0.2, 0.4, 0.6, 0.8 and 1, corresponding to the WGM order of $p=$1.

**Fig. 4:**
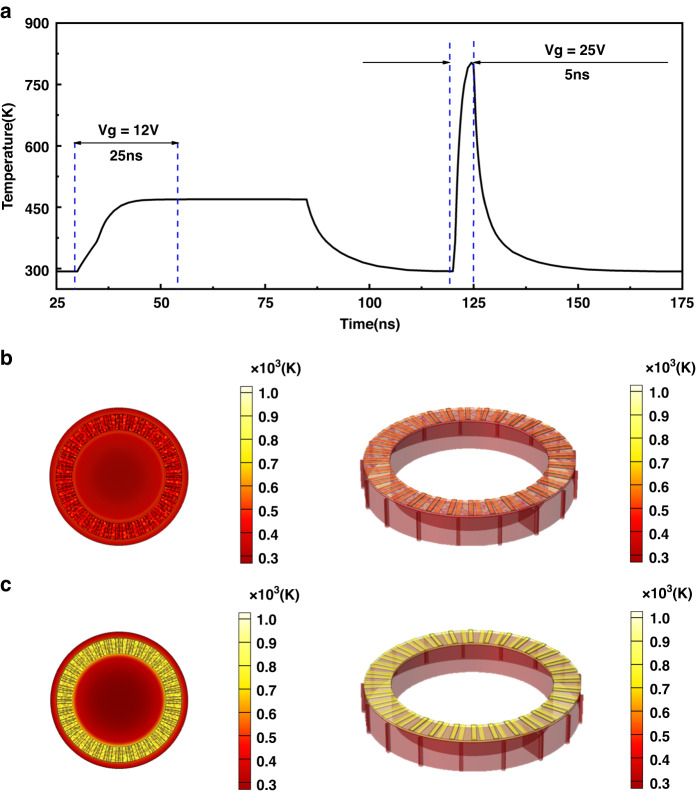
The electric-thermal transfer model of the GST225 temperature changes over time within the microring cavity. **a** Temperature of the GST225 dielectric layer in the ring cavity. The AD-AM GST225 dielectric layer is electrically heated above ${T}_{C}=$ 469 K, enabling it to convert into crystalline GST225 under ${V}_{g}=$1 V. The ${V}_{g}=$ 25 V is used to heat the crystalline GST225 above ${T}_{M}=$ 803 K. The melt-quenched (MQ) phase of GST225 results from subsequent quenching. The temperature distributions of the microring cavity at (**b**) 80 ns and (**c**) 125 ns where the color image presents the temperature values. The left and right columns display the temperature distributions at the *x*-*y* plane with $z=$ 650 nm and for the 3D structure, respectively

This OAM lasing is reconfigurable by reversibly switching the GST225 state between the crystalline and amorphous states. The GST225 dielectric possesses crystallization and melting temperatures of ${T}_{C}=$ 469 K and ${T}_{M}=$ 803 K, respectively. Amorphous GST225 crystallizes when it is heated over ${T}_{C}$ and remains below ${T}_{M}$^36^. By swiftly increasing the temperature above ${T}_{M}$, crystalline GST225 is re-amorphized. The WGM order can be engineered back and forth by the reversible phase transition of GST225, which provides reconfigurable OAM lasing. We use an electric-thermal transfer model to study how the GST225 temperature changes over time within the microring cavity (Fig. 4). In Table S1 of the Supplementary Information, the thermoelectric characteristics of the materials used in the simulation are provided. Figure 4a shows that the temperature in the as-deposited amorphous (AD-AM) GST225 increases above ${T}_{C}$ with the application of ${V}_{g}=$ 12 V for 25 ns. To completely crystallize GST225, we \perform a subsequent annealing process to keep the temperature above ${T}_{C}$ and below ${T}_{M}$ for ∼35 ns^51^. The crystallization ratio ($j$) of GST225 can be varied via electrical heating with time control; this, in turn gradually changes the refractive index of GST255 and causes the ring cavity resonant wavelength (OAM lasing mode) to continuously sweep across the spectra from 1544.5 to 1565.9 nm (Fig. 3b). The temperature of the crystalline GST225 layer is reduced to room temperature by disconnecting ${V}_{g}$ because the heat disperses into the surrounding air.

To reversibly shift the operating wavelength from 1565.9 to 1544.5 nm, a reverse re-amorphization (from crystalline to amorphous) is needed; here, the crystal lattice melts and then quenches to 293 K to prevent recrystallization of the atomic structure^52^. By applying ${V}_{g}=$ 25 V, we provide enough thermal energy to quickly raise the temperature above ${T}_{M}$ and melt the GST225. By shutting down ${V}_{g}$, the subsequent quick cooling quenches the melt in the amorphous phase. The forward wavelength tuning from 1544.5 to 1565.9 nm is again attained by adjusting ${V}_{g}$ back to 12 V. Figure 4b, c shows the 3D temperature distributions of the structure at 80 ns (crystallizing point) and 125 ns (melting point), respectively. Figure 4b shows that the entire AD-AM GST225 dielectric layer can exceed ${T}_{C}$ after 25 ns under ${V}_{g}=$ 12 V, whereas the peak temperature can be increased beyond ${T}_{M}$ after 5 ns under ${V}_{g}=$ 25 V. Video 1 records the entire process of reversibly tuning the emission spectra of the microring laser. Our proposed ring laser possesses exceptional performance for dynamically reconfigurable functions.

## Conclusion

In summary, an approach to impart phase change material (GST225) to microcavity tunable laser emission is demonstrated. Our proposed structure is based on combined index modulations and gain/loss at an EP, improving the degeneracy of the counterclockwise and clockwise whispering gallery modes and facilitating unidirectional power oscillation. A thermal-electric transfer model shows that the structural phase of GST225 can be changed back and forth in ~125 ns using an electrical voltage, resulting in a pronounced change in the refractive index of GST225. This can be used to dynamically tune the WGM order, thus controlling the emitted OAM lasing. This tunable OAM microlaser can facilitate exciting applications for OAM multiplexing in optical trapping and optical communications.

## Methods

### Simulation

With the finite element method, a numerical simulation of the parity-time symmetry vortex laser was performed using the fluctuating optics module (electromagnetic wave: frequency domain) of the commercial software COMSOL Multiphysics. We defined the background medium as a vacuum with a refractive index of 1. To calculate the characteristic frequency and Q-factor of the parity-time symmetry vortex laser during the phase transition of the GST225 layer and to extract the far-field characteristic vortex electromagnetic field distribution, we set up a group of parametric scans depending on the experimentally measured refractive index data of GST225 at different crystallization degrees for the simulation of the phase transition of the GST225 layer. We used a planar probe at the far field (*z* = 4800 nm) to extract the far-field characteristic vortex EMF distribution of the GST225 layer at different crystallization degrees of the parity-time symmetry vortex laser. The loss effect was neglected to simplify the simulation.

### Supplementary information

supporting information

Video1
