# Supplementary material for: Tunable parity-time symmetry vortex laser from a phase change material-based microcavity
Source: Microsyst Nanoeng. 2023 Nov 10;9:142. doi: 10.1038/s41378-023-00622-z (PMC10638240; doi:10.1038/s41378-023-00622-z)
Supplement: Supplementary file 1 — supporting information [file 41378_2023_622_MOESM1_ESM.docx]

Supplementary Information

Tunable Parity-Time Symmetry Vortex Laser from a Phase Change Material-based Microcavity

*Ying Su*^1^ *Hongji Fan*^1^ *Shitong Zhang* ^2^ *and Tun Cao*^1, *^

^1^School of Optoelectronic Engineering and Instrumentation Science, Dalian University of Technology, Dalian 116024, China

^2^School of Science and Letters, UC Davis, 2100 5th St, Davis, CA 95618, USA

* Corresponding author: *Tun Cao*, [caotun1806@dlut.edu.cn](mailto:caotun1806@dlut.edu.cn)

Table of Content

Note S1: Theoretical analysis of unidirectional laser oscillation at the EP

Fig S1: The tunable vortex laser emission from the ring cavity

Tab S1: List of parameters required for simulation

**Note S1. Theoretical analysis of unidirectional laser oscillation at the EP**

the refractive index equations as follow:

$n=\left\{\begin{matrix} n_{0}+in'' \\ n_{0}+n' \end{matrix}\right.\begin{matrix} 2\pi b/m<\theta<2\pi\left(b+1/4\right)/m \\ \text{ 2}\pi(b+3/8)/m<\theta<2\pi\left(b+5/8\right)/m \end{matrix}$ (s1)

where $n_{0}$ is the unperturbed part of the refractive index (effective index of GST225/InGaAsP stacked layers), $m$ the azimuthal number of the targeted WGM, $b$ integer number from {0, $m-$ 1}.

To investigate unidirectional power flow circulating inside the cavity at the exceptional point (EP) modification of index and loss/gain gratings ($n'=n''$), the semiconductor rate equations are adopt. The equations describe the linear and nonlinear competition of clockwise and counterclockwise whispering gallery modes (WGMs) in the microring cavity,

$\frac{dE_{cw}}{dt}=\gamma_{f}(1+i\alpha)[\xi_{1}N_{c}-1]E_{cw}-C_{1}E_{ccw}$ (s2)

$\frac{dE_{ccw}}{dt}=\gamma_{f}(1+i\alpha)[\xi_{2}N_{c}-1]E_{ccw}-C_{2}E_{cw}$ (s3)

$\frac{dN_{c}}{dt}=\gamma_{c}[\mu-N_{c}-\xi_{1}N_{c}\left|E_{cw}\right|^{2}-\xi_{2}N_{c}\left|E_{ccw}\right|^{2}]$ (s4)

where$E_{cw}(t)$and$E_{ccw}(t)$ are the slowly-varying normalized amplitudes of clockwise and counterclockwise propagating WGMs.$N_{c}$ is the carrier density, $\gamma_{f}$ is the field decay rate, $\gamma_{c}$ is the decay rate of the carrier population, $\alpha$ is the linewidth enhancement factor, $\mu$ is the normalized injection current, $\xi_{1}=1-s\left|E_{cw}\right|^{2}-c\left|E_{ccw}\right|^{2}$ and $\xi_{2}=1-c\left|E_{cw}\right|^{2}-s\left|E_{ccw}\right|^{2}$, $s$ and $c$ is the self- and cross-saturation coefficients. Linear mode coupling stems from the combined index and loss/gain gratings and is expressed by the parameters of $C_{1}$ and $C_{2}$ in the rate equations. In the lack of the alternate bilayer Cr/Ge and single-layer Ge gratings ($n'=n''=0$), $C_{1}$and $C_{2}$ do not completely disappear due to spurious backscattering in microring cavity while taking a small and indentical value,$C_{1}\text{=}C_{2}\text{=}C_{d}+iC_{c}$, where $C_{d}$ and $C_{c}$ are the dissipative and conservative couplings, respectively.^1,2^ When the two gratings are planted on the top of the microring, linear mode coupling is controlled by the relative strengths $n'$ and $n''$of the paired gratings. Particularly, when the paired grating strengths are indentical $n'=n''\neq0$, the linear coupling is unidirectional, e.g. $C_{2}/C_{1}\approx0$ corresponding to the EP operation of the microring laser.^3^ Nonlinear coupling of WGMs is caused by gain saturation. The modes saturate both each others and their own gains through the effects of carrier heating and spectral hole burning, which are contained in the *s* and *c>s* saturation parameters.^4^ Herein, grating effects in the carrier density is ignored since the standing-wave mode pattern exhibits a spatial period much smaller compared to the legnth of carrier diffusion. Therefore at the EP condition $C_{2}/C_{1}\approx0$, the counterclockwise WGM ($E_{ccw}$) can dominate over the the clockwise WGM ($E_{cw}$) and obtain stable and robust unidirectional laser oscillation.


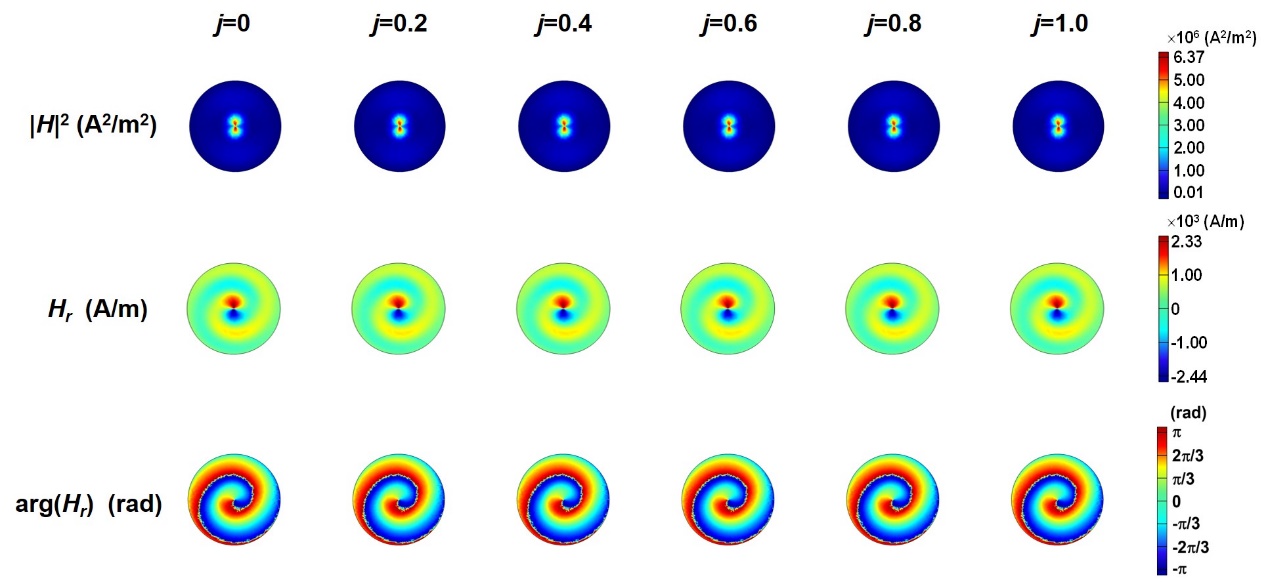


**Fig S1.** The tunable vortex laser emission from the ring cavity.The device maintains at the EP, having the indentical index modulation ($n'=n''\text{=}0.01$). The first row shows the distribution of $\left|H\right|^{2}$in the cross section at the middle of the microring (*z* = 0). The distributions of$H_{r}$and arg ($H_{r}$) in the cross section at far field (*z* = 4800 nm) are presented in the second, the third row, respectively. The resonant wavelengths of the ring cavity are λ = 1544.5, 1550.5, 1555.6, 1559.9, 1562.9 and 1565.9 nm for the different crystllisation ratios of *j* = 0, 0.2, 0.4, 0.6, 0.8 and 1.0, accordingly.

**Tab S1. List of parameters required for simulation**

|  | Special heat  capacity CS  (J / (Kg ⋅K)) | | Density  ρ(kg/m^3^ ) | Thermal  conductivity  k(W/(m⋅ k)) | Electrical  conductivity  sigma(S/m) | Relative  permittivity  epsilnr(1) | Seebeck  coefficient  S(V/K) |
| --- | --- | --- | --- | --- | --- | --- | --- |
| Cr | | 461^S9^ | 7190 ^S9^ | 69.1 ^S9^ | Temperature  Dependence  ^S10^ | Frequency  Dependence ^S12^ | Temperature  Dependence ^S12^ |
| Ge | | 301 ^S9^ | 5320 ^S9^ | 62.8 ^S9^ | 1740000^S9^ | Frequency  Dependence ^S12^ | Temperature  Dependence ^S12^ |
| InGaAsP | | Temperature  Dependence^S12^ | 5316^S9^ | Temperature  Dependence ^S12^ | Temperature  Dependence ^S12^ | Frequency  Dependence ^S12^ | Temperature  Dependence ^S12^ |
| GST225 | | Temperature  Dependence^S11^ | 6200^S8^ | Temperature  Dependence  ^S5-S7^ | Temperature  Dependence ^S5-S7^ | Frequency  Dependence ^S12^ | Temperature  Dependence ^S12^ |

**References**

S1 Sorel, M.; Laybourn, P. J. R.; Scire, A.; Balle, S.; Giuliani, G.; Miglierina, R.; Donati, S. Opt. Lett. 27, 1992-1994 (2002).

S2 Sorel, M.; Giuliani, G.; Scire, A.; Miglierina, R.; Donati, S.; Laybourn, P. J. R. IEEE J. Quantum Electron. 39, 1187-1195 (2003).

S3 Longhi, S.; Feng, L. Opt. Lett. 39, 5026-5029 (2014).

S4 Van der Sande, G.; Gelens, L.; Tassin, P.; Scire, A.; Danckaert, J. J. Phys. B. 41, 095402 (2008).

S5 Fallica, R.; Battaglia, J. L.; Cocco, S.; Monguzzi, C.; Teren, A.; Wiemer , C.; Varesi, E.; Cecchini, R.; Gotti, A.; Fanciulli, M. J. Chem. Eng. Data 54, 1698 (2009).

S6 Endo, R.; Maeda, S.; Jinnai, Y.; Lan, R.; Kuwahara, M.; Kobayashi, Y.; Susa, M. Japan. J. Appl. Phys. 49, 065802 (2010).

S7 Lyeo, H. K.; Cahill, D. G.; Lee, B. S.; Abelson, J. R.; Kwon, M. H.; Kim, K. B.; Bishop, S. G.; Cheong, B. Appl. Phys. Lett. 89, 151904 (2006).

S8 Harnsoongnoen, S.; Sa-Ngiamsak, C.; Siritaratiwat, A. Int. J. Mod. Phys. B 23, 3625 (2009).

S9 Dieter, G. E. Metallic Materials Specification Handbook. E & F.N.Spon LTD (1972).

S10 Deyoung, T.; Arajs, S.; Anderson, E. ANTIFERROMAGNETISM AND ELECTRICAL RESISTIVITY OF CHROMIUM-IRIDIUM ALLOYS. Am. Inst. Phys. (1972).

S11 Silva, H.; Bakan, G.; Cywar, A.; Williams, N.; Dirisaglik, F.; Henry, N. Crystallization of silicon microstructures through rapid self-heating for high-performance electronics on arbitrary substrates. Nanosci. Nanotechnol. Lett. 23, 225201 (2012).

S12 Weast, R. C.; Astle, M. J.; Beyer, W. H.; Company, C. R.; Selby, S. M.; Lide, D. R. Crc handbook of chemistry and physics. Am. J. Med. Sci. (1982).
